# A practical integrated radiomics model predicting intensive care hospitalization in COVID-19

**DOI:** 10.1186/s13054-021-03564-y

**Published:** 2021-04-14

**Authors:** Chiara Giraudo, Giovanni Frattin, Giulia Fichera, Raffaella Motta, Roberto Stramare

**Affiliations:** Department of Medicine – DIMED, University of Padova, Via Giustiniani 2, 35100 Padua, Italy

It has been widely demonstrated that radiological imaging significantly contributes to diagnosing and monitoring pulmonary and systemic involvement of patients affected by COVID-19 using different techniques like chest X-ray (CXR), Computed Tomography (CT), and Magnetic Resonance Imaging [1–3]. Recently, several authors proposed the application of advanced imaging tools including machine learning and radiomics for COVID-19. For instance, Chandra et al. developed an automatic screening method based on radiomic features and Wang et al. used radiomics to distinguish COVID-19 from other viral infections [4, 5]. Moreover, Ferreira Junior et al. [6], using a publicly available cohort, demonstrated that radiomics not only correlates with the etiologic agent of acute infections but also supports the short-term risk stratification of COVID-19 patients. Inspired by these interesting results, we developed and tested a CXR-based radiomics integrated model including demographics, first-line laboratory and clinical findings collected at admission and we assessed the predicting role of such model for Intensive Care Unit (ICU) hospitalization and overall outcome. We retrospectively examined CXR at admission of 203 patients hospitalized in our tertiary center for COVID-19 (positive at RT-PCR) (Table 1). Eighteen patients deceased; 56 patients were treated in ICU and 147 in COVID-19 wards only. One radiologist with 10 years of experience in thoracic imaging, segmented the lungs of each patient as illustrated in Fig. 1 using an open source software (3D Slicer, www.slicer.org). The manual segmentation was performed by the segment editor and paint tools avoiding the inclusion of hilar and cardiac shadows. The radiomics extension was applied for the extraction of 33 features of first and second order: first-order statistics, gray level co-occurrence matrix, and gray level run length matrix. Factor analysis allowed the selection of five features highly correlating: maximum, kurtosis, inverse variance, cluster shade, and run length non-uniformity normalized (Fig. 1). The logistic regression analysis demonstrated that among the five radiomic features and the clinical and laboratory variables, only inverse variance, run length non-uniformity normalized, and C-reactive protein levels were significant predictors of ICU hospitalization (each, *p* < 0.05). None of the examined variables played a significant role in predicting the overall outcome (*p* > 0.05, each).

**Table 1:** Characteristics of the examined population

Characteristics at admission	Entire cohort	Patients treated in ICU	Patients treated in Covid-19 wards
Age (years) (mean ± SD)	67.6 ± 14	68.8 ± 10	67.2 ± 15.6
Gender (female/male)	60/143	10/46	50/97
Fever (> 37.5 C) (yes/no)	178/25	51/5	127/20
Status (alive/deceased)	185/18	49/7	136/11
Red blood cells count (× 10^12^ L^−1^) (mean ± SD)	4.5 ± 0.6	4.4 ± 0.5	4.5 ± 0.6
Hemoglobin (g/l) (mean ± SD)	13 ± 2	13 ± 2	13 ± 2
White blood cells count (× 10^9^ L^−1^) (mean ± SD)	7 ± 4	8 ± 4	6.8 ± 4
Lymphocytes count (× 10^9^ L^−1^) (mean ± SD)	1.1 ± 0.9	0.8 ± 0.4	1.1 ± 1
C-reactive protein (mg/L^−1^) (mean ± SD)	80 ± 69	116 ± 88	65 ± 54

**Fig. 1:**
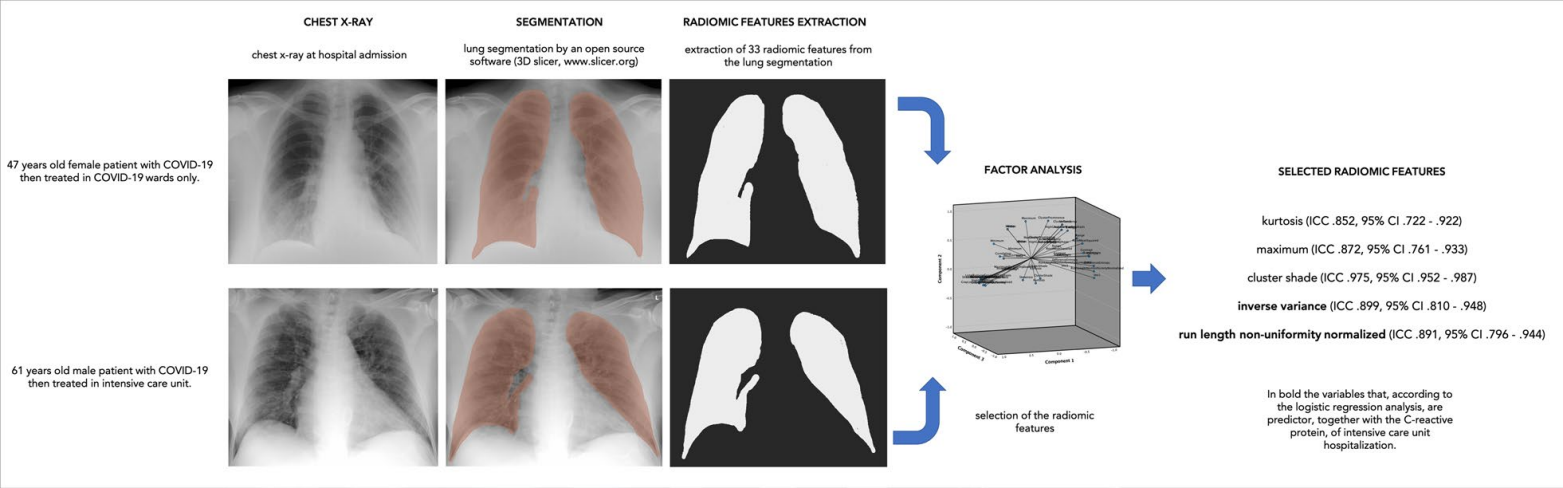
Graphic representation of the development of the proposed radiomics model demonstrating the segmentation and extraction of radiomic features from chest X-ray at admission of COVID-19 patients and the factor analysis that led to the selection of five highly correlating variables. The robustness of the proposed model is shown by the excellent (> .750) intraclass correlation coefficient values of the selected variables

To evaluate the robustness of the proposed method, a second reader, a medical student with 1 year of experience in diagnostic imaging repeated all segmentations and data extraction. The intraclass correlation coefficient analysis (two-way random effects, consistency, two raters) showed excellent results (each > 0.750; Fig. 1) indicating that the technique is reproducible also by readers without a long-time expertise. Thus, our preliminary results demonstrate that a rapid model based on two radiomics feature and a basic inflammatory index, collected at admission, can predict ICU hospitalization. Our findings are partially in agreement with Ferreira Junior et al. [6] who showed that radiomics is a predictor not only of progression but also of the overall outcome. The difference regarding the outcome could be due to the low number of deceased patients in our cohort and the lower number of investigated features. A multicenter study with a more homogeneous distribution of the outcome may provide new insights. Regarding the number of features, we wanted to propose a practical radiomics model which could be placed side by side to routine reporting in clinical practice. It has to be addressed that several studies used CT for COVID-19 similar models since this technique is more accurate in characterizing disease severity and extension. Nevertheless, CXR still plays a dominant role especially as first approach and our results show that significant quantitative information can be extracted also from this diagnostic tool. Our study is limited by the retrospective study design. We call for future prospective research possibly correlating radiomics with semi-quantitative visual scores.

## Data Availability

The datasets used and/or analysed during the current study are available from the corresponding author on reasonable request.
